# Identification and Characterization of a Novel Protein Disulfide Isomerase Gene (*MgPDI2*) from *Meloidogyne graminicola*

**DOI:** 10.3390/ijms21249586

**Published:** 2020-12-16

**Authors:** Zhongling Tian, Zehua Wang, Maria Munawar, Jingwu Zheng

**Affiliations:** 1 Laboratory of Plant Nematology, Institute of Biotechnology, College of Agriculture & Biotechnology, Zhejiang University, Hangzhou 310058, China; tzl@zju.edu.cn (Z.T.); maria.munawar@yahoo.com (M.M.); 2 Institute of Insect Science, College of Agriculture and Biotechnology, Zhejiang University, Hangzhou 310058, China; 382732003@zju.edu.cn; 3 Key Lab of Molecular Biology of Crop Pathogens and Insects, Ministry of Agriculture, Hangzhou 310058, China

**Keywords:** *Meloidogyne graminicola*, protein disulfide isomerase, cell death, RNAi, host-pathogen interaction

## Abstract

Protein disulfide isomerase (PDI) is a multifunctional enzyme that catalyzes rate-limiting reactions such as disulfide bond formation, isomerization, and reduction. There is some evidence that indicates that PDI is also involved in host-pathogen interactions in plants. In this study, we show that the rice root-knot nematode, *Meloidogyne graminicola*, has evolved a secreted effector, MgPDI2, which is expressed in the subventral esophageal glands and up-regulated during the early parasitic stage of *M. graminicola*. Purified recombinant MgPDI2 functions as an insulin disulfide reductase and protects plasmid DNA from nicking. As an effector, MgPDI2 contributes to nematode parasitism. Silencing of *MgPDI2* by RNA interference in the pre-parasitic second-stage juveniles (J2s) reduced *M. graminicola* multiplication and also increased *M. graminicola* mortality under H_2_O_2_ stress. In addition, an *Agrobacterium*-mediated transient expression assay found that MgPDI2 caused noticeable cell death in *Nicotiana benthamiana*. An intact C-terminal region containing the first catalytic domain (a) with an active motif (Cys-Gly-His-Cys, CGHC) and the two non-active domains (b and b′) is required for cell death induction in *N. benthamiana*. This research may provide a promising target for the development of new strategies to combat *M. graminicola* infections.

## 1. Introduction

Protein disulfide isomerase (PDI) is a member of the thioredoxin superfamily and is a multifunctional enzyme that catalyzes rate-limiting reactions such as disulfide bond formation, isomerization, and reduction in the endoplasmic reticulum (ER) [1,2]. In addition, PDIs also act as molecular chaperones which assist the refolding of denatured proteins without disulfide bonds, such as citrate synthase [3,4], and their molecular structure and biological activity have been well characterized in yeast, humans, and some parasitic protozoa [5,6].

A typical PDI consists of four domains, including two thioredoxin-like catalytic domains (a and a′) each containing a Cys-Gly-His-Cys (CGHC) active site motif and two non-catalytic domains (b and b′) [7]. Moreover, the PDI contains a signal peptide at the N-terminus and the ER retention signal is located in the small C-terminal domain (c). While some PDI gene members do not contain ER sequences, it has been noted that the amino acid composition of some ER sequences differs [8]. PDI also plays an important role in host-pathogen interactions [9]. For example, studies have reported higher expression of a PDI gene (LmPDI) in the parasitic *Leishmania major* indicating that PDI expression is correlated with infection and might be a causative agent [10]. A PDI gene (PpPDI1) from *Phytophthora parasitica* contributes to plant infection and causes necrosis of tobacco leaves when transiently expressed in *Nicotiana benthamiana* [11]. HsPDI, a candidate effector, is involved in the interaction between *Heterodera schachtii* and host plants and plays an important role in protecting *H. schachtii* from the harm of reactive oxygen species (ROS) released by host plants [12].

*Meloidogyne graminicola* is one of the most important plant-parasitic nematodes (PPN) that attacks rice. It is also a quarantine pest that has caused substantial destruction in most rice-growing countries [13,14,15]. Studies of the interaction between rice and *M. graminicola* revealed that rice enacts defense responses during nematode infections. It was observed that basal defense in rice increased during β-aminobutyric acid-induced resistance against *M. graminicola*, which includes the expression of genes related to hormones, ROS generation, and callose deposition [16]. Recent studies suggested that the effectors secreted by *M. graminicola* can interfere with the host immune responses such as the effectors MgGPP and MgMO237 observed to promote the *M. graminicola* parasitism by inhibiting cell death and manipulating rice basal immunity, respectively [17,18].

Recently, a PDI in *M. graminicola* (MgPDI1) was reported to be involved in protection against oxidative damage as a nematode effector [19]. In this study, we characterized a second PDI from *M. graminicola* (MgPDI2) via analyses of the expression pattern, enzymatic activity, physiological effects associated with gene silencing and necrosis induction in *N. benthamiana*. Together, our data indicate that MgPDI2 contributes to *M. graminicola* infection.

## 2. Results

### 2.1. Identification and Sequence Analysis of the MgPDI2 Gene from M. graminicola

A 1573 bp cDNA of *MgPDI2* (GenBank accession number MT517333) was cloned from *M. graminicola*. The *MgPDI2* gene includes an open reading frame (ORF) of 1569 bp encoding a putative protein of 522 amino acids with a predicted molecular size of 58.7 kDa. According to the SignalP program, MgPDI2 contains a secretion signal peptide of 20 amino acids at its N-terminus, indicating that MgPDI2 might be secreted into the host tissue by *M. graminicola*.

**Figure 1:**
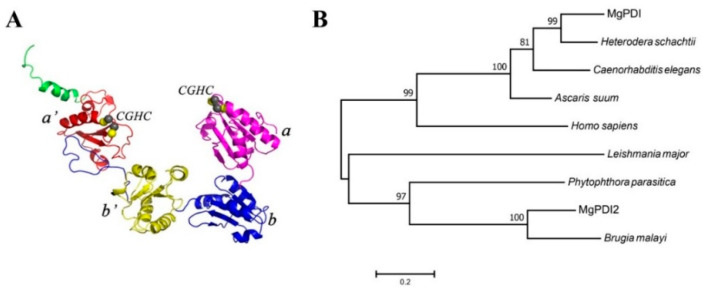
Sequence analysis of MgPDI2. (**A**) The tertiary structure of MgPDI2. (**B**) Molecular phylogenetic analysis of protein disulfide isomerase (PDI) proteins from different organisms based on PDI-deduced amino acid sequences. The numbers indicated on the nodes are the bootstrap values for each cluster based on 1000 permutations.

NCBI Conserved Domains search program analysis revealed that MgPDI2 contained four conserved thioredoxin domains (a, b, b′, a′) (Figure 1). In the 3-D tertiary structure, MgPDI2 is predicted to have a shape of a twisted “U”, in which the a and a′ domains are found on the ends while the b and b′ domains form the base of the U (Figure 1). In addition, the a and a′ domains each contain one active site which is characterized by a Cys-Gly-His-Cys (CGHC) catalytic motif (Figure 1A). Phylogenetic analysis based on PDI-deduced amino acid sequences of different organisms revealed the distant relationship of two MgPDI proteins from *M. graminicola*. MgPDI and MgPDI2 are in two different clades. MgPDI2 grouped with PDIs from *P. parasitica* and *Brugia malayi* while MgPDI grouped with PDIs from *H. schachtii* and *C. elegans* (Figure 1B).

### 2.2. MgPDI2 Is Expressed in the Subventral Esophageal Glands and Was Up-Regulated during the Early Parasitic Stage of M. graminicola

**Figure 2:**
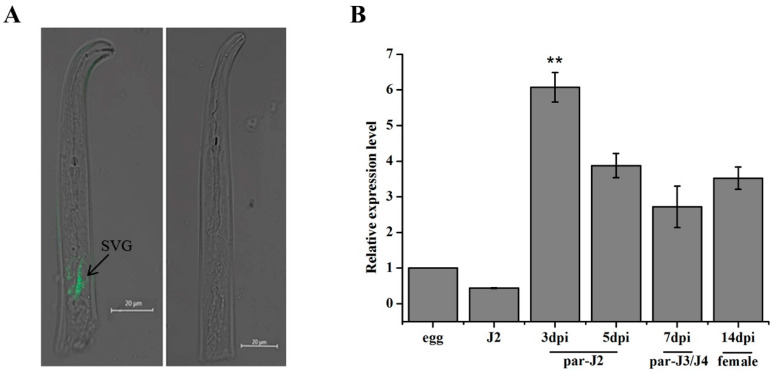
Expression patterns of *MgPDI2* in *M. graminicola***.** (**A**) Localization of *MgPDI2* by *in situ* hybridization. Right: antisense cDNA probe; left: sense cDNA probe. (**B**) Expression pattern of *MgPDI2* in five different developmental stages of *M. graminicola*: par-J2, par-J3 and par-J4, parasitic second-, third- and fourth-stage juveniles. **: significant differences based on Tukey’s test (*p* < 0.01)

In order to determine the tissue localization of *MgPDI2* in pre-parasitic *M. graminicola* juveniles, *in situ* hybridization was performed. Strong green fluorescent signals were observed in the subventral gland cells of the pre-parasitic second-stage juveniles (J2s) after the Digoxin (DIG)-labelled antisense probe of *MgPDI2* specifically hybridized with transcripts, while no signal was detected when using the labeled sense probe (Figure 2A). qRT-PCR was used to further analyze the transcriptional expression pattern of *MgPDI2* during different developmental stages of *M. graminicola*. The transcription levels in sedentary stages of nematode were relatively high, reaching its maximum level at 3 dpi relative to actin gene expression (Figure 2B).

### 2.3. Recombinant Expression and Purification of MgPDI2

**Figure 3:**
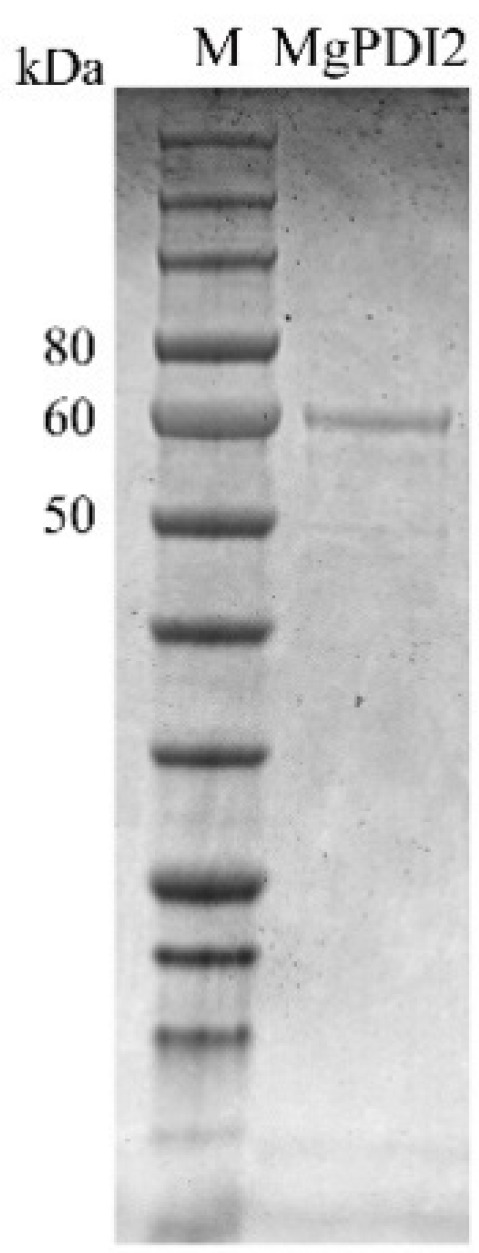
The 12% SDS-PAGE analysis of the purified MgPDI2 expressed in *Escherichia coli* BL21 (DE3). The ladder used as size standards for the gel consists of the following molecular weight bands: 250, 150, 100, 80, 60, 50, 40, 30, 25, 20, 15, and 10 kDa.

To obtain the MgPDI2 protein, recombinant plasmids were transformed into *Escherichia coli* BL21 (DE3). MgPDI2 was successfully expressed at 20 °C. Thrombin was used to cleave the fusion proteins in order to exclude possible effects of His-Thrombin tag. The recombinant MgPDI2 protein was approximately 58 kDa after purification, which was the expected size (Figure 3). The final concentration of MgPDI2 was adjusted to 1 mg/mL after thrombin removal for enzymatic activities detection.

### 2.4. Enzymatic Activities of MgPDI2 Protein

**Figure 4:**
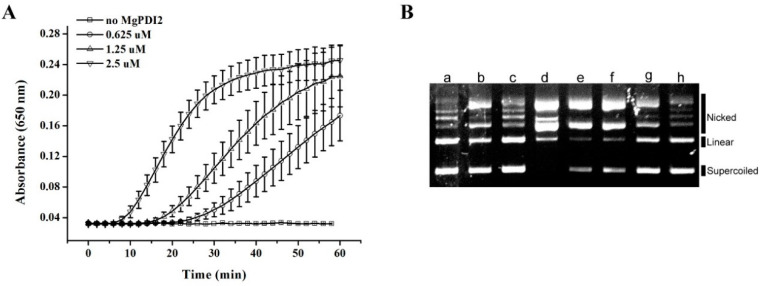
Biological activity of recombinant MgPDI2. (**A**) Dithiothreitol (DTT)-mediated insulin reduction by recombinant MgPDI2. (**B**) Potential of recombinant MgPDI2 to protect super-coiled DNA from cleavage in a mixed-function oxidase (MFO) system. Lanes: a, pGEM-T without any treatment; b, pGEM-T incubated with 1.65 mM DTT; c, pGEM-T incubated with 16.5 mM FeCl3; d, pGEM-T incubated with the MFO system; e–h, pGEM-T incubated with the MFO system and different concentrations (0.1, 1, 10, and 100 μg/mL, respectively) of purified MgPDI2. The nicked, linear, and super-coiled forms of pGEM-T are shown from the top to the bottom. Numerical values are reported in Table 1.

Protein disulfide isomerase (PDI) is a member of the thioredoxin superfamily. Thioredoxin activities are often assessed by detection in a protein reduction assay and, alternatively, through an antioxidant activity assay. As the reduction of insulin disulfide bonds is a standard method to assess PDI reductive assay, we used a bovine insulin reduction assay that detects the reduction of insulin by measuring increasing turbidity at OD650. The reduction of bovine insulin disulfide bonds increased as the MgPDI2 protein concentrations increased (Figure 4A), and the negative control reaction lacking MgPDI2 showed no insulin disulfide bond reduction (Figure 4A). A plasmid DNA nicking assay was performed to assess the ability of MgPDI2 to function as an antioxidant relevant to DNA protection. MgPDI2 protects supercoiled DNA (a pGEM-T plasmid) from oxidative damage, which causes a nicked form of the DNA (Figure 4B). The extent of DNA damage in the mixed-function oxidase (MFO) system was indicated by two distinct forms of pGEM-T (nicked form, upper bands; super-coiled form, lower bands) after electrophoretic separation on a gel. The super-coiled form of the plasmid was converted into the nicked form (Figure 4B, lane d) when it was incubated with the MFO system. When MgPDI2 was included in the MFO system, less nicking was detected (Figure 4B, lanes e to h), suggesting that MgPDI2 defends DNA against metal-mediated oxidative damage to DNA. Furthermore, this inhibition of nicking by MgPDI2 occurred in a dosage-dependent manner, as observed by the results of the mixture incubated with different concentrations of MgPDI2 (Figure 4B, Table 1).

In the lane row, alphabetical characters indicate the number of lanes in Figure 4B. Values represent percentages of the plasmid in the nicked, linear, and supercoiled forms, derived from their intensity. Lane a shows spontaneous nicking of the plasmid in buffer; lane b shows nicking in the presence of dithiothreitol; lane c shows nicking in the presence of FeCl_3_; lane d shows nicking in the presence of the MFO system; lanes e-h show nicking in the presence of the MFO system and different concentrations of purified MgPDI2.

### 2.5. MgPDI2 Is Involved in Parasitism

**Figure 5:**
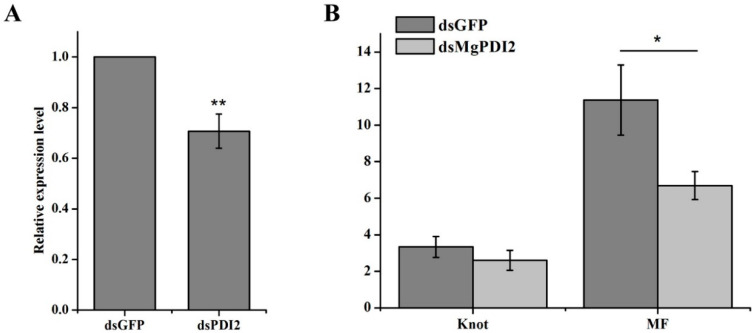
Effect of *MgPDI2* expression on rice susceptibility to *M. graminicola* infection. (**A**) *MgPDI2* expression in nematode after treatment with dsRNA targeting *MgPDI2*. Asterisks indicate significant differences based on Tukey’s test (*: *p* < 0.05; **: *p* < 0.01). (**B**) Average numbers of knots and the MF values at 15 dpi.

To analyze whether *MgPDI2* plays a role in *M. graminicola* parasitism, in vitro RNAi of *MgPDI2* was performed. The qRT-PCR (Figure 5A) results showed a decrease in the transcript abundance of *MgPDI2* in the J2s that were soaked with *MgPDI2* dsRNA. Then, rice roots were infected by J2s that were soaked in dsRNA targeting *MgPDI2* or *GFP* and the nematode multiplication factor (MF) ((number of egg masses × number of eggs per egg mass) ÷ nematode inoculum level = MF) at 15 dpi was determined. Our analysis showed that the MF was reduced significantly in plants infected with juveniles treated with dsRNA against *MgPDI2* (MF = 6.69) compared to *GFP* (MF = 11.37) (Figure 5B).

### 2.6. MgPDI2 Expression Is Induced by H_2_O_2_ and Increases H_2_O_2_ Tolerance

**Figure 6:**
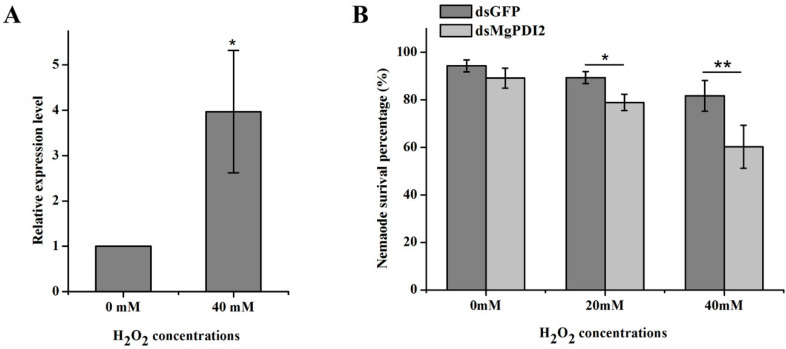
*MgPDI2* expression is triggered by H_2_O_2_ and increases H_2_O_2_ tolerance. (**A**) The relative mRNA expression levels of *MgPDI2* in response to the H_2_O_2_ stress. (**B**) Effect of *MgPDI2* silencing on H_2_O_2_ stress tolerance. Asterisks indicate significant differences based on Tukey’s test (*: *p* < 0.05; **: *p* < 0.01).

qRT-PCR was used to analyze the expression of *MgPDI2* in response to H_2_O_2_. A significant increase in transcript abundance of *MgPDI2* was found in juveniles that were exposed to 40 mM H_2_O_2_ compared to 0 mM H_2_O_2_ for 30 min (Figure 6A). We examined the mortality rate of *MgPDI2* or *GFP* dsRNA-treated J2s after soaking them in different concentrations (0, 20, 40 mM) of H_2_O_2_ (Figure 6B). We found that a significantly lower percentage of J2s with silenced *MgPDI2* survived after exposure to 20 mM and 40 mM H_2_O_2_ compared to *GFP*-silenced controls.

### 2.7. MgPDI2 Induces Strong Necrotic Responses in N. benthamiana

**Figure 7:**
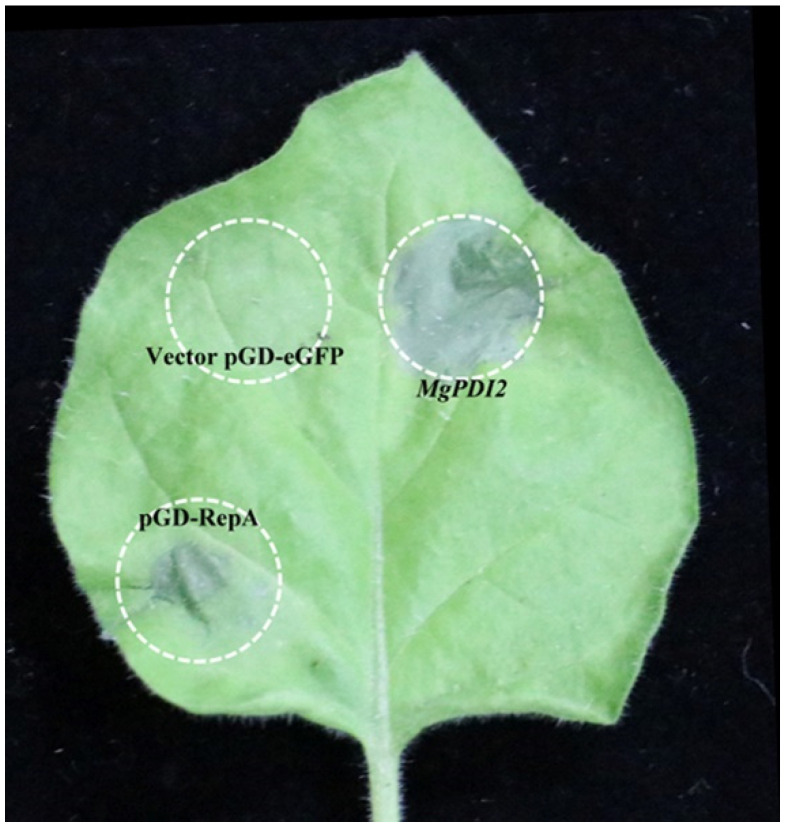
Transient expression of MgPDI2 induces cell death in *N. benthamiana*. Infiltration of *Agrobacterium tumefaciens* strain EHA 105 carrying a *MgPDI2* expression construct into leaves of 6-week-old *N. benthamiana* plants. Empty pGD-eGFP vector was used as a negative control, while pGD-RepA was used as a positive control.

In order to determine if *MgPDI2* can work as a pathogenicity factor that induces cell death in *N. benthamiana*, an *Agrobacterium*-mediated transient expression assay in *N. benthamiana* leaves was performed. Transient expression of *MgPDI2* caused noticeable cell death in *N. benthamiana* 48 h after Agro-infiltration, while the empty vector pGD-eGFP construct did not induce cell death (Figure 7). Western blotting indicated that the MgPDI2:pGD-eGFP fusion protein was expressed in the leaves of *N. benthamiana*, thereby supporting a role for MgPDI2 in inducing host plant necrosis.

### 2.8. Functional Domains of MgPDI2 Are Required for Cell Death Induction

**Figure 8:**
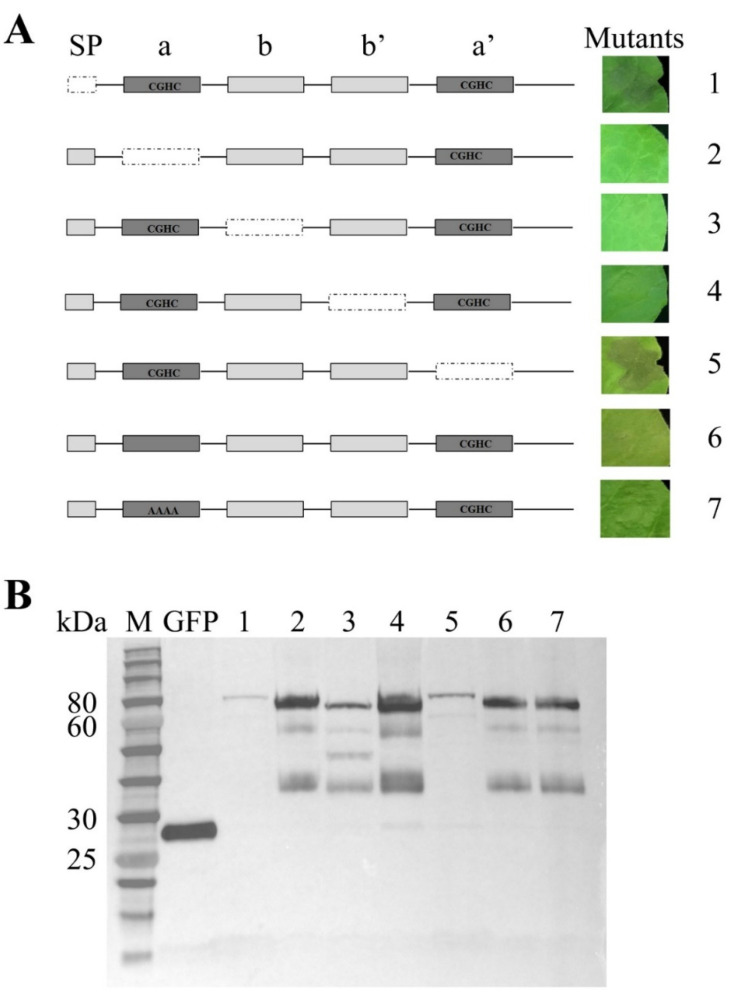
Determination of MgPDI2 domains required for cell death induction. (**A**) Schematic views of MgPDI2 and deletion mutants are shown on the left. The deletion mutants of MgPDI2 were transiently expressed in *N. benthamiana* leaves. (**B**) Western blot analysis of the MgPDI2 deletion series. The anti-GFP primary antibody was used to detect the accumulation of fused proteins in the leaves of *N. benthamiana* plants. 1: signal peptide deletion mutant; 2: “a” deletion mutant; 3: “b” deletion mutant; 4: “b′” deletion mutant; 5: “a′” deletion mutant; 6: CGHC in domain “a” deletion mutant; 7: CGHC in domain “a” replaced by AAAA mutant. The ladder used as size standards for the gel consists of the following molecular weight bands: 250, 150, 100, 80, 60, 50, 40, 30, 25, 20, 15, and 10 kDa. The presence of numerous bands might be due to the poor antibody specificity.

In order to identify which domain(s) of MgPDI2 are required for cell death induction, several deletion mutants were constructed to assess domain-specific effects on cell death induction by *Agrobacterium tumefaciens*-mediated transient expression in *N. benthamiana*. Our results depicted that deletion mutants lacking the a, b, and b′ domains, respectively, lost the ability to induce cell death, whereas the deletion mutants lacking the signal peptide or a′ can still induce cell death in *N. benthamiana* (Figure 8A). However, the mutants with the CGHC catalytic motif in domain a deleted or replaced with alanine residues AAAA both lost the ability to induce cell death (Figure 8A). The results of our Western blot confirmed that these mutated fusion proteins were expressed in the leaves of *N. benthamiana* (Figure 8B), while the presence of numerous bands might be due to the poor antibody specificity. Thus, we demonstrated the roles of MgPDI2 domains with respect to host plant necrosis.

## 3. Discussion

In this study, we found that MgPDI2 encodes a typical PDI in the endoparasitic nematode *M. graminicola*. MgPDI2 is involved in *M. graminicola* pathogenicity of host plants and it can also induce cell death in *N. benthamiana*. These results indicated that *MgPDI2* might play a role in *M. graminicola* virulence.

In the 3-D tertiary structure prediction, MgPDI2 contained four conserved thioredoxin domains (a, b, b′, a′) which were found to be arranged in the shape of a twisted “U” in which the a and a′ domains were located on the ends while the b and b′ domains formed the rounded base of the U (Figure 1A). This thioredoxin domain arrangement is typical of members of the PDI family [20]. In addition, the a and a′ domains each contain one active site which is characterized by a Cys-Gly-His-Cys (CGHC) catalytic motif (Figure 1A). This CGHC motif is the most common conserved motif in PDIs [20], and the catalytic motifs appear to be highly conserved in the various eukaryotes. Therefore, MgPDI2 is annotated as a typical PDI. Phylogenetic analysis revealed that MgPDI and MgPDI2 are in two different clades (Figure 1B). MgPDI2 grouped with PDIs from *P. parasitica* and *Brugia malayi*, while MgPDI grouped with PDIs from *Heterodera schachtii* and *Caenorhabditis elegans*. The distant relationship between MgPDI and MgPDI2 indicates that these two proteins are distinct in amino acid sequences. During this study, it was also noted that the functions of both proteins are also completely different, MgPDI can suppress reactive oxygen species (ROS) production in *N. benthamiana* induced by flg22 (unpublished data), while MgPDI2 can induce strong necrotic responses in *N. benthamiana* which further supports the unique characteristics of MgPDI2 compared to MgPDI.

*In situ* hybridization results indicated that *MgPDI2* was expressed in esophageal gland cells of pre-J2, which is thought to be one of the origins of nematode effector secretion (Figure 2A). These results suggested that MgPDI2 can be secreted into host tissues by *M. graminicola* to facilitate infection [21]. In addition, our qRT-PCR data showed that the *MgPDI2* transcription levels in sedentary stages of nematode were relatively high, particularly in the early parasitic stages, reaching its maximum at 3 dpi (Figure 2B). This expression pattern suggested that *MgPDI2* might play a role in *M. graminicola* infection. The spatial and temporal expression pattern of *MgPDI2* is very similar to the expression pattern of *MgPDI* and *HsPDI* in *Heterodera schachtii* [12,19].

Like the recombinant MgPDI protein from *M. graminicola*, recombinant MgPDI2 was highly expressed at 20 °C (Figure 3) [19]. Previous studies reported that PDIs have enzymatic activities relevant to oxidization, isomerization, and reduction [2,22]. Here, we tested the reduction and antioxidant activities of MgPDI2. For the reductive assays of MgPDI2, a classical insulin reduction assay was performed, and the results indicated that MgPDI2 can reduce insulin disulfide bonds (Figure 4A). This reductive activity is consistent with that of other PDIs as observed in previous studies [2,19]. However, compared to MgPDI of *M. graminicola*, the reduction activity of MgPDI2 is likely stronger than that of MgPDI as the reduction of disulfide bonds using 0.625 μM recombinant MgPDI2 is better than that of 2.5 μM recombinant MgPDI [19]. To assess the antioxidant activity of MgPDI2, we used an MFO system to generate thiol radicals that damage DNA to assess how MgPDI2 was able to protect DNA integrity. Our results suggested that MgPDI2 can protect plasmid DNA from nicking in a dosage-dependent manner (Figure 4B). Tian et al. [19] demonstrated that MgPDI also possessed this kind of antioxidant activity. It was reported that as PDI is a member of the thioredoxin superfamily, PDI plays a key role in several pivotal antioxidant and redox regulation processes [22]. Thioredoxin-mediated protection of DNA from oxidative damage was also demonstrated in *Haemonchus contortus* [23]. The two above enzymatic activities are consistent with the functions of typical PDIs which contain four conserved thioredoxin-like domains. Therefore, we confirmed that MgPDI2 is a functional PDI.

To analyze whether *MgPDI2* plays a role in the parasitism process of *M. graminicola*, in vitro RNAi targeting *MgPDI2* was performed. We found that pre-parasitic J2s treated with ds*MgPDI2* reduced to the MF 6.69 compared to an MF of 11.37 when treated with ds*GFP* (Figure 6B). RNAi can be used to investigate the function of pathogenicity factors in both sedentary and migratory nematodes [24]. Our results are consistent with the conclusions drawn in previous studies, such as, Habash et al. [12] reported that silencing *HsPDI* by RNAi reduced the number of *H. schachtii* eggs [12]; Tian et al. [19] demonstrated that *MgPDI* silencing caused a reduction in the MF of *M. graminicola*. Together, these findings suggest that PDI is essential in the reproduction of parasitic nematodes.

Similar to *MgPDI*, 40 mM H_2_O_2_ can induce the expression of *MgPDI2* (Figure 6A) [19]. Moreover, it is noted that both 20 and 40 mM H_2_O_2_ can significantly increase the mortality rate of *MgPDI2* dsRNA-treated J2s (Figure 6B). These results suggest that MgPDI2 plays a role in protecting J2s from the impact of the exogenous H_2_O_2_. Habash et al. [12] and Dubreuil et al. [24] also reported that *HsPDI* from *H. schachtii* and peroxiredoxins from *Meloidogyne incognita* increased tolerance to exogenous H_2_O_2_, respectively.

Some pathogen effectors can elicit cell death in *N. benthamiana*. For example, it is demonstrated that the effector protein RBP-1 from *Globodera pallida* can be recognized by the NB-LRR protein Gpa2 to elicit cell death in *N. benthamiana* [25]. PDI1 of *P. parasitica* (PpPDI1) and *G. pallida* (GpPDI1) also triggered cell death in *N. benthamiana* [11,26]. In this study, we found that the secreted protein MgPDI2 from *M. graminicola* induced noticeable cell death in *N. benthamiana* 48 h after Agro-infiltration (Figure 7), indicating that MgPDI2 is sufficient for inducing cell death in *N. benthamiana*. However, deletion mutants lacking a, b, and b′ domains lost the ability to induce cell death. These data indicate that an intact C-terminus containing domains a, b, and b′ is required for activity. Furthermore, deleting or replacing the CGHC catalytic motif in domain a with alanine residues prevented cell death induction in *N. benthamiana*, indicating that the first active CGHC motif is also essential for cell death induction by MgPDI2 (Figure 8A). These results indicated that the first catalytic domain (a), particularly the CGHC catalytic motif in domain a, and the two non-active domains (b and b′) are required for cell death induction in *N. benthamiana*, while the signal peptide and the domain a′ have no role in inducing cell death in *N. benthamiana*. However, it is unusual that PDI, a ROS scavenger, caused cell death in *N. benthamiana*. One possible explanation is that only a single effector was examined in the experiments and an Agrobacterium-mediated transient expression assay was carried out in a nematode-free system meaning “in the absence of other nematode effectors”.

As a nematode effector expressed in *N. benthamiana*, MgPDI2 may be recognized by *N. benthamiana*’s immune receptors, and trigger cell death which is a characteristic of a hypersensitive response (HR). In spite of that, when MgPDI2 is secreted into host roots through the stylet after *M. graminicola* natural parasitism, it may not be as strong to cause cell death in the rice root. Besides, MgPDI2 may not be recognized as a threat by the immune receptors of rice that trigger effector-triggered immunity (ETI) -type defense. Another explanation is that the nematode might have evolved an unknown mechanism which can interfere with the ETI-type defense to successfully parasitize rice [26]. In the nematode-host interactions, other effectors of *M. graminicola* may suppress cell death induced by MgPDI2. For example, the *M. graminicola* effector, MgGPP, was reported to suppress cell death [17]. Further work is required to determine the role of MgPDI2 in the natural parasitism of *M. graminicola*.

Consequently, our study proposes that MgPDI2 contributes to *M. graminicola* infection of rice based on our characterization of the gene expression, transcript localization, biochemical characterization, and gene silencing-mediated physiological assays. In addition, MgPDI2 might contribute to the activation of host defense responses due to its ability to induce cell death in *N. benthamiana*.

## 4. Materials and Methods

### 4.1. Plant Growth Conditions

The rice line used in this work was *Oryza sativa* cv. “Nipponbare”, seeds were germinated on wet filter paper for 8 d at 28 °C, transferred to potting soil in a growth chamber, and were grown under a 16 h/8 h light/dark photoperiod at 28 °C/26 °C and relative humidity of 75%. Tobacco (*Nicotiana benthamiana*) was grown in the greenhouse under 16 h/8 h light/dark photoperiod at 28/26 °C, with 60% relative humidity.

### 4.2. M. graminicola Culture Conditions

The *M. graminicola* isolate ZJJH was maintained on rice (*Oryza sativa* cv. “Nipponbare”) in potting soil in a grow chamber under a 16 h/8 h light/dark photoperiod at 28 °C/26 °C [27].

#### Cloning and Sequence Analyses

The full-length cDNA sequence of *MgPDI2* was obtained by rapid amplification of cDNA ends using the SMART RACE cDNA Amplification kit (Clontech, Carlsbad, CA, USA) according to the manufacturer’s instructions based on *M. graminicola* transcriptome data [28]. All primers used in this study are listed in Table 2. An online protein structure homology-modeling server, SWISS-MODEL, was used to predict MgPDI2 tertiary structure. The signal peptide of MgPDI2 was predicted using the SignalP-5.0 Server (http://www.cbs.dtu.dk/services/SignalP/). The conserved domain search for MgPDI2 was performed using the NCBI CD search program (https://www.ncbi.nlm.nih.gov/Structure/cdd/wrpsb.cgi) [29]. The phylogenetic tree was constructed using maximum likelihood algorithms in MEGA 6, and PDI amino acid sequences from different organisms are described as the following: MgPDI (MH392200), *Phytophthora parasitica* (XP_008914616.1), *Ascaris suum* (ERG84937.1), *Caenorhabditis elegans* (NP_491995.1), *Homo sapiens* (CAA89996.1), *Leishmania major* (AAN75008.1), *Heterodera schachtii* (ANA05342.1), and *Brugia malayi* (XP_001899304.1).

### 4.3. In Situ Hybridization

The DIG-labelled sense and antisense RNA probes (402-bp) were synthesized using the DIG RNA labeling mix (Roche, Mannheim, Germany). The specific primers that were used are listed in Table 2. Approximately 8000 freshly hatched second-stage juveniles (J2s) were fixed and *in situ* hybridization was performed following the modified protocol of De Boer et al. [23]. After hybridization, the nematodes were examined by light microscopy using a Nikon ECLIPSE Ni microscope (Nikon, Tokyo, Japan).

### 4.4. Developmental Expression Analysis

Total RNA derived from *M. graminicola* nematodes at different developmental stages (par-J2: 3dpi-5dpi; par-J3/J4: 7dpi; female: 14dpi) were extracted using the TRIzol method (Invitrogen, Carlsbad, CA, USA) according to the manufacturer’s instructions. The cDNA was synthesized using the ReverTra Ace qPCR RT kit (Toyobo, Osaka, Japan). qRT-PCR specific to MgPDI2 was performed with the MgPDI2-RT-F/ MgPDI2-RT-R (Table 2) primer pair and actin genes of *M. graminicola* were amplified with Mg-ACT-Q-F/ Mg-ACT-Q-R primers [28,30]. The qRT-PCR reactions were performed on a CFX Connect real-time system (BIO-RAD, Hercules, CA, USA) using SYBR Premix Ex Taq II (Tli RNaseH Plus) (Takara, Tokyo, Japan). Three technical replicates for each reaction were performed and three independent experiments were performed with the following conditions: 95 °C for 60 s; 40 cycles of 95 °C for 15 s and 60 °C for 30 s. The relative changes in gene expression were determined using the 2^−ΔΔCT^ method [31].

### 4.5. Expression, Purification, and Validation of Recombinant MgPDI2

The cDNA fragments encoding MgPDI2 without the signal peptide were amplified by PCR primers containing restriction sites (Table 2). Products of the PCR assembly were ligated into the pET-32a vector (Novagen, San Diego, CA, USA) using T4 DNA ligase (Promega, Madison, WI, USA). The resulting recombinant vector was validated by PCR product sequencing and was transformed into *Escherichia coli* BL21 (DE3) competent cells (TaKaRa, Dalian, China) for protein expression. Transformed *E. coli* was cultured at 37 °C while shaking at 180 rpm in lysogeny broth (LB) containing ampicillin (50 µg/mL) until the optical density at 600 nm (OD600) reached 0.5. Afterward, isopropyl-b-D-thiogalactoside (IPTG) was added to a final concentration of 0.5 mM. The induction was then carried out at 20 °C and 180 rpm shaking. After induction for 24 h, the bacterial cells were pelleted by centrifugation, the bacterial pellet was suspended and lysed by His TALONTM xTractor buffer (Clontech, Mountain View, CA, USA), then treated with DNAse I (Qiagen). The recombinant protein was purified using His TALON™ Gravity Columns (Clontech, Mountain View, CA, USA) according to the manufacturer’s instructions. Recombinant protein expression was verified on a 12% SDS-PAGE. Thrombin (Sigma, MO, USA) was used to cleave the His-Thrombin Tag from recombinant MgPDI2 on His TALON™ Gravity Columns (Clontech, Mountain View, CA, USA) to purify the recombinant proteins, and p-amino benzamidine-agarose (Sigma, St. Louis, MO, USA) was applied to bind thrombin after cleavage. Finally, the supernatant was collected, and protein concentration was measured by absorbance at 280 nm and then stored at 80 °C [32].

### 4.6. MgPDI2 Reductive Assay

For the PDI reductive assay, the MgPDI2 enzyme activity to reduce bovine insulin was adapted from a modified method [33,34]. Reaction mixtures (200 µL total) included 100 mM Tris-Cl (pH 6.8), 2 mM ethylene diaminetetraacetic acid (EDTA), 0.13 mM insulin from bovine pancreas (Sigma, St. Louis, MO, USA), 0.33 mM dithiothreitol (DTT), and increasing concentrations (ranging from 2.5 to 10 µM) of purified MgPDI2 protein. The turbidity of the reaction mixture was monitored by measuring the absorbance increase at 650 nm using a Microplate Reader (Thermo, Hudson, NH, USA). The reduction of insulin by DTT was recorded in a solution without MgPDI2 as a negative control.

### 4.7. Protective Effect of MgPDI2 against Oxidative Damage by the MFO System

The ability of MgPDI2 to protect DNA from oxidative damage was determined by the method used by Tian et al. [19]. Briefly, the mixed-function oxidase (MFO) system, consisting of 1.65 mM DTT, 16.5 mM FeCl_3_, and different concentrations of MgPDI2 (from 0.1 to 100 µg/mL) were pre-incubated at 37 °C for 2.5 h. pGEM-T easy plasmid DNA (500 ng, Promega, Madison, WI, USA) was then added and incubated for 1 h at 37 °C. Nicking of DNA was evaluated by ethidium bromide staining after electrophoresis separation in 0.8% agarose gels [35,36].

### 4.8. RNAi and Infection Assay

Approximately 500 bp of DNA fragments were amplified by PCR from both *GFP* and *MgPDI2*. The forward and reverse primers contained T7 promoter sequences at their 5′ end for in vitro RNA synthesis (Table 2). Double-stranded RNA (dsRNA) was synthesized and purified using the T7 RiboMAXTM Express kit (Promega, Madison, WI, USA) according to the manufacturer’s instructions. The concentration of RNA was determined by measuring absorbance at 260 nm.

RNAi soaking was performed using the modified method [37,38]. 20,000 Freshly hatched J2s of *M. graminicola* were soaked in the dsRNA solution (2 mg mL^−1^ dsRNA, 3 mM spermidine, 50 mM octopamine, and 0.05% gelatin, adjusted with 0.25 × M9 buffer) for 36 h at room temperature in the dark on a rotator. Nematodes soaked in solutions with dsRNA targeting GFP were used as controls. Afterward, for each reaction, about 8000 J2s were used by qRT-PCR to evaluate the level of *MgPDI2* silencing. Approximately 500 J2s were used to determine the ability to survive under H_2_O_2_ stress. All remaining J2s were used for plant infection assays.

For the infection assays, Pluronic F-127 (PF-127) (Sigma-Aldrich, St. Louis, MO, USA) gel was used as previously reported [39,40], 2-week-old rice seedlings (*Oryzae sativa* cv Nipponbare) were individually transplanted in a standard tube (25 mm × 95 mm) containing 10 mL Pluronic F-127 medium, inoculated with 80 J2s soaked in dsRNA targeting *MgPDI2* or *GFP*, and incubated in a growth chamber under 16 h/8 h light/dark photoperiod at 28 °C/26 °C for 15 d. Seven replications were included in each experiment and each experiment was repeated at least twice [41].

At 15 dpi, rice roots were stained with acid fuchsin and the number of eggs was counted after dissecting the stained galls under a microscope [42]. To determine the reproductive potential of *M. graminicola*, the nematode multiplication factor (MF) ((number of egg masses × number of eggs per egg mass) ÷nematode inoculum level = MF) was calculated.

### 4.9. MgPDI2 Expression during H_2_O_2_ Stress

To check *MgPDI2* gene expression under H_2_O_2_ stress, 5000 J2s were incubated in 40 mM H_2_O_2_ for 30 min and washed in sterile tap water. J2s incubated in sterile tap water were used as a control. The expression of *MgPDI2* was quantified by qRT-PCR as described above.

#### Effect of MgPDI2 Depletion on Nematode Survival Following H_2_O_2_ Stress

Around 200 freshly hatched J2s were incubated overnight in a solution of *MgPDI2* dsRNA or *GFP* dsRNA as described above and then washed in tap water. Afterward, the J2s were incubated in 0, 20, and 40 mM H_2_O_2_ for 30 min. The percentage of dead J2s was calculated for each treatment.

### 4.10. Agrobacterium-Mediated Transient Expression

The coding sequence of *MgPDI2* was cloned into the pGD-eGFP vector, and the *MgPDI2*:pGD-eGFP construct was transformed into *A. tumefaciens* strain EHA 105. *Agrobacterium*-mediated transient expression was carried out as described by Tian et al. [19]. *A. tumefaciens* suspensions were adjusted with infiltration buffer to an OD600 of 1.0 and mixed with P19 (an RNA silencing inhibitor) at 1:1. After incubation for 2 h at room temperature, the *A. tumefaciens* strains carrying the constructs were injected into the abaxial side of 6-week-old *N. benthamiana* plant leaves using a 1 mL hypodermic syringe without a needle. Infiltrated plants were incubated in the growth chamber (16 h light, 8 h dark at 25 °C) for 5 d. The empty pGD-eGFP vectors were used as a negative control. The primer sequences are given in Table 2.

### 4.11. Western Blot Analysis

The *N. benthamiana* leaf samples were harvested 48 h after infiltration. Total protein was extracted from powdered plant tissue using a phenol solution (0.5 M Tris pH 9.4, 50 mM EDTA, 0.7 M sucrose, 0.1 M KCl containing 2% ß-mercaptoethanol and complete protease inhibitors (Roche)). The protein concentration was measured using a Bradford assay following the manufacturer’s instructions (Bio-Rad). 0.1 mg protein of each sample was separated on Tris-Glycine SDS-PAGE gels and transferred to nitrocellulose membranes using an iBlot (semi-) dry blotting system from Life Technologies. The blotting membrane was blocked with 5% BSA (bovine serum albumin) in TBST solution (50 mM Tris-HCl, pH 7.5, 150 mM NaCl, 0.05% Tween 20) for 1 h at room temperature. The membrane was incubated with 1:3000 diluted anti-GFP primary antibody (Sigma) for 3 h at room temperature. The membrane was washed with TBST solution and incubated with a 1:3000 dilution of anti-mouse IgG antibody (Sigma). The signal was detected using an AP conjugate substrate kit (Bio-Rad).

### 4.12. Statistical Analysis

All statistical analyses were performed in SPSS Statistics 20.0 software (IBM). Data had a normal distribution and are presented as means ± standard deviation (SD). All data were analyzed by ANOVA (one-way) with a Tukey-test, with a significance threshold of *p* < 0.05.

## Figures and Tables

**Table 1:** Potential of recombinant MgPDI2 to protect super-coiled DNA from cleavage in a mixed-function oxidase (MFO) system.

Lane	a	b	c	d	e	f	g	h
Recombinant MgPDI2 (μg/mL)	0	0	0	0	0.1	1	10	100
Nicked form (%)	44.39	61.06	51.91	89.98	85.74	82.62	59.37	41.72
Linear form (%)	26.49	17.54	22.92	10.02	4.72	5.23	17.18	25.91
Supercoiled form (%)	29.12	21.41	25.17	0.00	9.53	12.15	23.45	32.37

**Table 2:** Primer sequences used in this study.

Primer Name	Primer Sequences (5′-3′)	Use
MgPDI2-F	GTCATGATCTCCTTTTCTGTTC	open reading frame (ORF) verification
MgPDI2-R	CAATGAAAAGAACAACAGGGAGG	ORF verification
MgPDI2-BamhI	CGCGGATCCAGTGAAAAGGTTTCTCCTAC	Vector construction
MgPDI2-XholI	CCGCTCGAGAGCTCAGTATGGCCCTCCTC	Vector construction
MgPDI2-RT-F	TGATGAGGGACGTGCTGACT	qRT-PCR
MgPDI2-RT-R	CTCCACCAAAAATGACGGC	qRT-PCR
Mg-ACT-Q-F	AAGATCCTCACTGAGCGTGGTTAC	qRT-PCR
Mg-ACT-Q-R	CTTGACCGTCAGGCAATTCATAGC	qRT-PCR
MgPDI2-P	TTATGATTCTGCCGTTGC	In situ hybridization
MgPDI2-AP	CAAAGAAATGAGACGAACAGC	In situ hybridization
MgPDI2-T7-P	TAATACGACTCACTATAGGGTTATGATTCTGCCGTTGC	In situ hybridization
MgPDI2-T7-AP	TAATACGACTCACTATAGGGCAAAGAAATGAGACGAACAGC	In situ hybridization
MgPDI2-dsRNA-P	CCAAGGAGTCCCCTGATTTT	dsRNA
MgPDI2-dsRNA-AP-T7	TAATACGACTCACTATAGGGTCTCAGCATCAGAAAGACCAG	dsRNA
MgPDI2-dsRNA-P-T7	TAATACGACTCACTATAGGGCCAAGGAGTCCCCTGATTTT	dsRNA
MgPDI2-dsRNA-AP	TCTCAGCATCAGAAAGACCAG	dsRNA
MgPDI2-GFP-P	TCTACAAATCTATCTCTGGATCCATGATCTCCTTTTCTGTTCT	Vector construction
MgPDI2-GFP-AP	TCGCCCTTGCTCACCATGGATCCAAGCTCAGTATGGCCCTCCT	Vector construction
MgPDI2-NSP-P	TCTACAAATCTATCTCTGGATCCATGAGTGAAAAGGTTTCTCCTAC	MgPDI2 mutants
MgPDI2-NSP-AP	TCGCCCTTGCTCACCATGGATCCAAGCTCAGTATGGCCCTCCT	MgPDI2 mutants
MgPDI2-a-AP	AACATTCTCTTCCTCTTCAA	MgPDI2 mutants
MgPDI2-a-P	AAGAGGAAGAGAATGTTAAGAAGAAGACTGGACCTCC	MgPDI2 mutants
MgPDI2-b-AP	TGGAGGTCCAGTCTTCTTCT	MgPDI2 mutants
MgPDI2-b-P	AGAAGACTGGACCTCCAAGAATTCCTCTTGTTTCAGA	MgPDI2 mutants
MgPDI2-b′-AP	TTGGCTAAATTCTGAAACAA	MgPDI2 mutants
MgPDI2-b′-P	TTTCAGAATTTAGCCAAGATGGAAAGTTGAAGCCACA	MgPDI2 mutants
MgPDI2-a′-AP	TGTTTGTCCCAATCCTCGGG	MgPDI2 mutants
MgPDI2-a′-P	GAGGATTGGGACAAACATGACTCTGGTGGTAAAGAAG	MgPDI2 mutants
MgPDI2-Del-AP	CCACGGAGCATAGAACTCTA	MgPDI2 mutants
MgPDI2-Del-P	AGTTCTATGCTCCGTGGAAGGCATTAGCTCCAGAATA	MgPDI2 mutants
MgPDI2-AAAA-AP	GCCTTGGCGGCGGCGGCCCACGGAGCATAGAACTCTA	MgPDI2 mutants
MgPDI2-AAAA-P	GCCGCCGCCGCCAAGGCATTAGCTCCAGAATAT	MgPDI2 mutants

All primers are listed in 5′-3′ orientation.

## Abbreviations

MgPDI2	Protein Disulfide Isomerase 2
CGHC	Cys-Gly-His-Cys
dpi	days post-infection
pre-J2	pre-parasitic second-stage juvenile
